# High-Throughput Screening for Biomarker Discovery

**DOI:** 10.1155/2015/108064

**Published:** 2015-04-28

**Authors:** Tavan Janvilisri, Haruo Suzuki, Joy Scaria, Jenn-Wei Chen, Varodom Charoensawan

**Affiliations:** ^1^Department of Biochemistry, Faculty of Science, Mahidol University, Bangkok 10400, Thailand; ^2^Graduate School of Science and Engineering, Yamaguchi University, Yamaguchi 753-8511, Japan; ^3^Department of Veterinary & Biomedical Sciences, College of Agriculture & Biological Sciences, South Dakota State University, Brookings, SD 57006, USA; ^4^Center of Infectious Disease and Signaling Research, National Cheng Kung University, Tainan 701, Taiwan

Biomarkers are indicators of disease occurrence and progression. They can be used to assess normal biological and pathogenic processes or pharmacologic responses to a therapeutic intervention and, in some cases, may serve as potential drug and prognostic targets. In the postgenomic era, the omics techniques have been applied and served as high-throughput screening for identification of potential biomarkers for pathogenesis of several diseases including cancers and metabolic diseases, as well as infectious diseases. These biomarkers would lead to the improved understanding of the mechanism of pathogenesis and might be clinically useful as the molecular targets for better diagnosis, prognosis, and treatment.

In this special issue, there are six articles: two reviews and four original research articles. The first review article focuses on the application of proteomics in the discovery of cancer biomarkers, while the others focus specifically on biomarkers in nasopharyngeal carcinoma derived through omics approaches. Two research articles focus on protein biomarkers in cholangiocarcinoma using proteomics, one of which attempted to search for serum diagnostic markers and the other investigated the biomarkers for metastasis. Finally, the other two original papers address the relationship between gene polymorphic and miRNA biomarkers and diseases. Thus, the articles in this special issue represent a broad spectrum of experimental approaches and areas of investigation and demonstrate a wide array of molecular biomarker research.

In “Novel Serum Biomarkers to Differentiate Cholangiocarcinoma from Benign Biliary Tract Diseases Using a Proteomic Approach,” T. Janvilisri et al. identified proteins in the serum that can potentially discriminate the patients with cholangiocarcinoma from individuals with benign biliary tract diseases through proteomic approach using highly stringent analysis with cross-validation. They identified potential serum molecular markers that are worth further validation and could be useful for distinguishing between these two diseases with similar appearance, leading to better therapeutic measures.

In “Proteomics in Cancer Biomarkers Discovery: Challenges and Applications,” R. M. Sallam provided us with a perspective review that summarizes the potential use of proteomics approach to identify molecular targets in cancer research with examples of three of the most studied cancers including lung, breast, and ovarian cancers. This review describes the appreciation of proteomics in cancer research, which makes us understand tumor biology better, facilitates the development of biomarkers, and, most importantly, makes us move towards bedside applications in cancer management.

In “Analysis of Serum MicroRNAs as Potential Biomarker in Coronary Bifurcation Lesion,” Y. Liu et al. investigated circulating miRNAs for coronary bifurcation lesion in order to identify potential biomarkers. They performed miRNA profiling using microarray to screen the serum miRNAs profiles of patients with coronary bifurcation lesion and coronary nonbifurcation lesions. The miRNAs identified in this study may play a part in pathogenesis of coronary bifurcation lesion and could serve as novel biomarkers for the diagnosis and prognosis of this disease in the future.

In “Comparative Proteomic Analysis of Human Cholangiocarcinoma Cell Lines: S100A2 as a Potential Candidate Protein Inducer of Invasion,” K. Wasuworawong et al. attempted to identify metastatic protein markers in cholangiocarcinoma through protein expression profiling of highly invasive cell line, KKU-M213, and lowly invasive one, KKU-100. They proposed that S100A2 could potentially be a key protein involved in the progression of cholangiocarcinoma and may pose as a biomarker as well as a novel therapeutic target for this type of cancer.

In the review “Omics-Based Identification of Biomarkers for Nasopharyngeal Carcinoma,” T. Janvilisri provided an overview of the discovery of molecular biomarkers for nasopharyngeal carcinoma through the emerging omics technologies including genomics, miRNA-omics, transcriptomics, proteomics, and metabolomics. A large number of potential biomarkers for this disease related to various pathophysiological states have been discussed. This review article, thus, could be a reference point for downstream research in the field of nasopharyngeal carcinoma biomarkers.

In “Polymorphisms in* C-Reactive Protein* and* Glypican-5* Are Associated with Lung Cancer Risk and* Gartrokine-1* Influences Cisplatin-Based Chemotherapy Response in a Chinese Han Population,” S. Zhang et al. investigated polymorphic variations in seven genes including* CRP*,* GPC5*,* ACTA2*,* AGPHD1*,* SEC14L5*,* RBMS3,* and* GKN1*, which have previously been associated with lung cancer. They found the relationship between* CRP* and* GPC5* variants and risks for lung cancer. Variation in* GKN1* has also been shown to correlate to chemotherapy response in the Chinese population.

At present, the biomarker data are blooming through the omics research. It will be a challenge to merge a vast amount of data and acquire biological meanings to resolve a daunting conundrum of different diseases. Integrative approaches together with further validations will be necessary. The editorial team hopes that this special issue will be useful for investigators in the field.

